# Supplementary material for: Modulating Electron Density of Boron–Oxygen Groups in Borate via Metal Electronegativity for Propane Oxidative Dehydrogenation
Source: Materials (Basel). 2024 Jun 12;17(12):2868. doi: 10.3390/ma17122868 (PMC11205058; doi:10.3390/ma17122868)
Supplement: Supplementary file 1 [file materials-17-02868-s001.zip › materials-3028063-supplementary.pdf]

materials

Article

Modulating Electron Density of Boron–Oxygen Groups in Borate via Metal Electronegativity for Propane Oxidative Dehydrogenation

Panpan Li [1,2], Yongbin Yao [1,2], Shanshan Chai [1,2], Zhijian Li [1,2], Fan Xue [1,2] and Xi Wang [1,2,*]

1 Key Laboratory of Luminescence and Optical Information, Ministry of Education, School of Physical Science and Engineering, Beijing Jiaotong University, Beijing 100044, China; 19118030@bjtu.edu.cn (P.L.); yyb0321shzu@163.com (Y.Y.); 20118011@bjtu.edu.cn (S.C.); 23111524@bjtu.edu.cn (Z.L.); 22121615@bjtu.edu.cn (F.X.)
2 Tangshan Research Institute of Beijing Jiaotong University, Tangshan 063000, China
* Correspondence: xiwang@bjtu.edu.cn

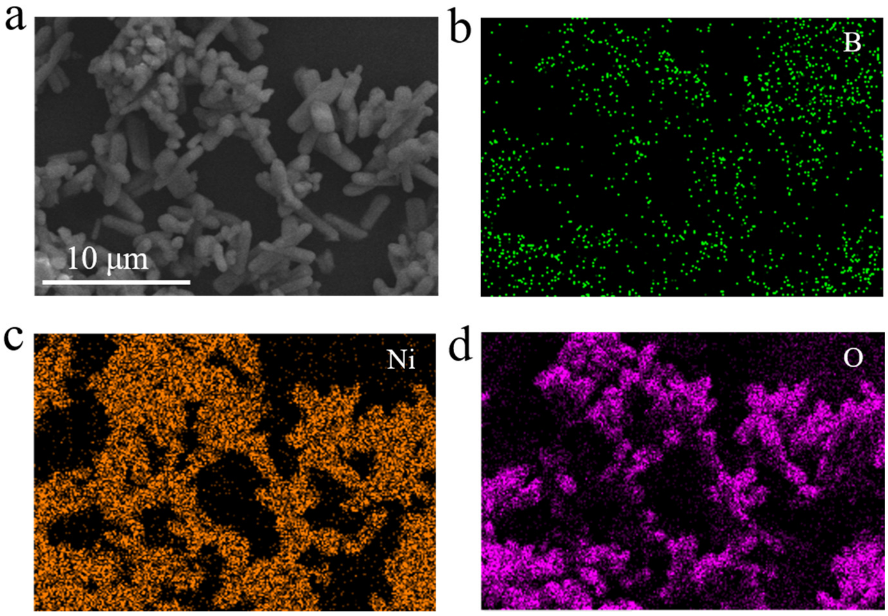

Figure S1. (a) Scanning electron microscope (SEM) images of catalyst NiBOx-1000, the corresponding EDS mapping of (b) B, (c) Ni, (d) O.

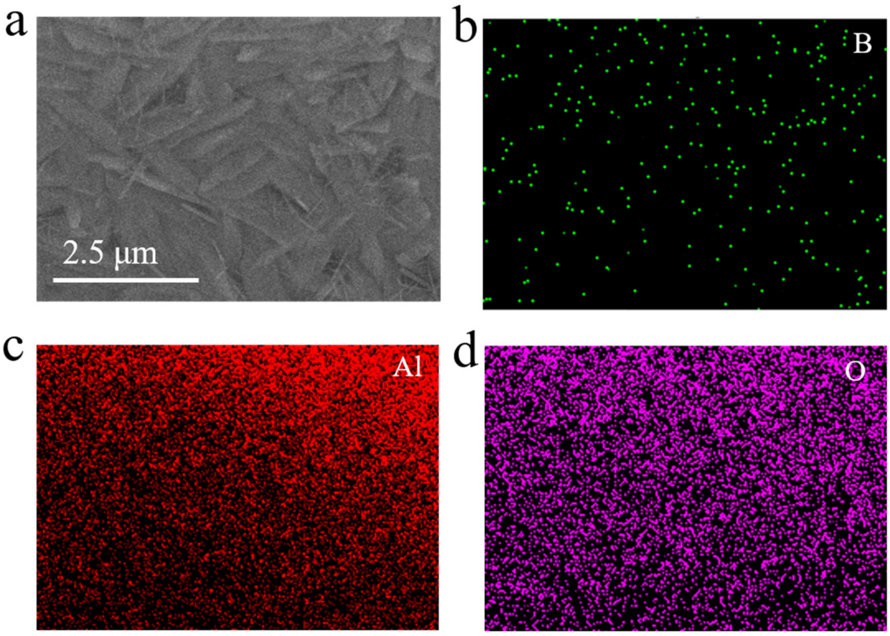

Figure S2. (a) Scanning electron microscope (SEM) images of catalyst AlBOx-1000, the corresponding EDS mapping of (b) B, (c) Al, (d) O.

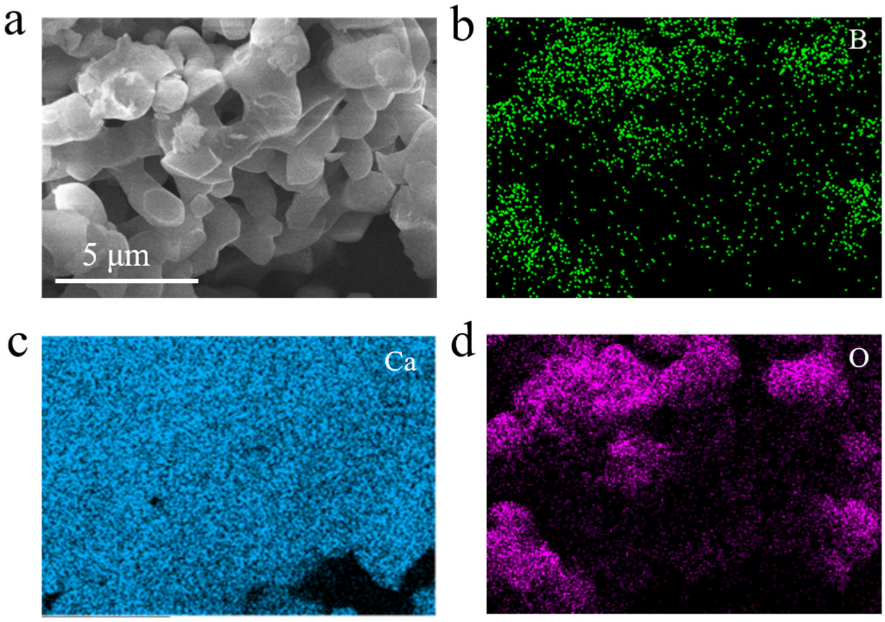

Figure S3. (a) Scanning electron microscope (SEM) images of catalyst CaBOx-1000, the corresponding EDS mapping of (b) B, (c) Ca, (d) O.

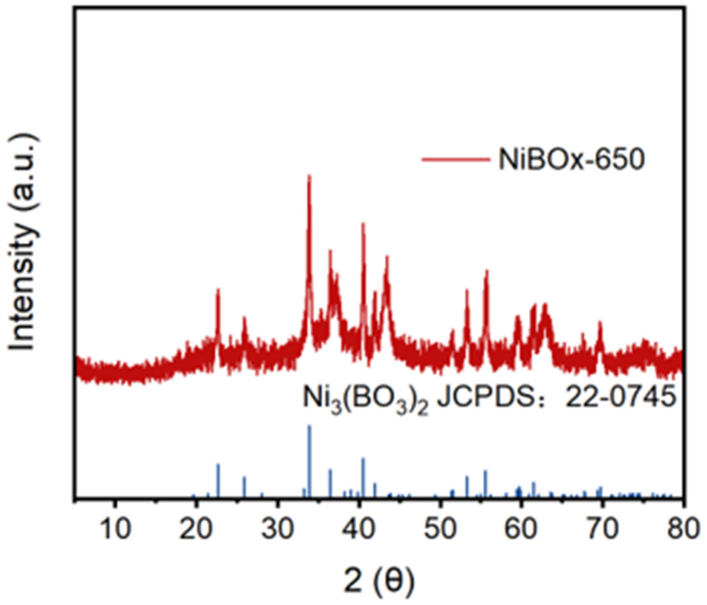

Figure S4. X-ray diffraction patterns of NiBOx-650.

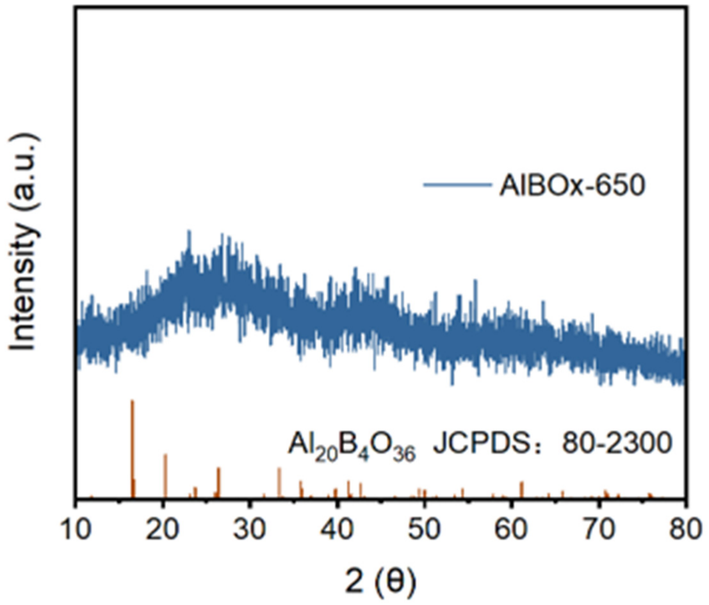

Figure S5. X-ray diffraction patterns of AlBOx-650.

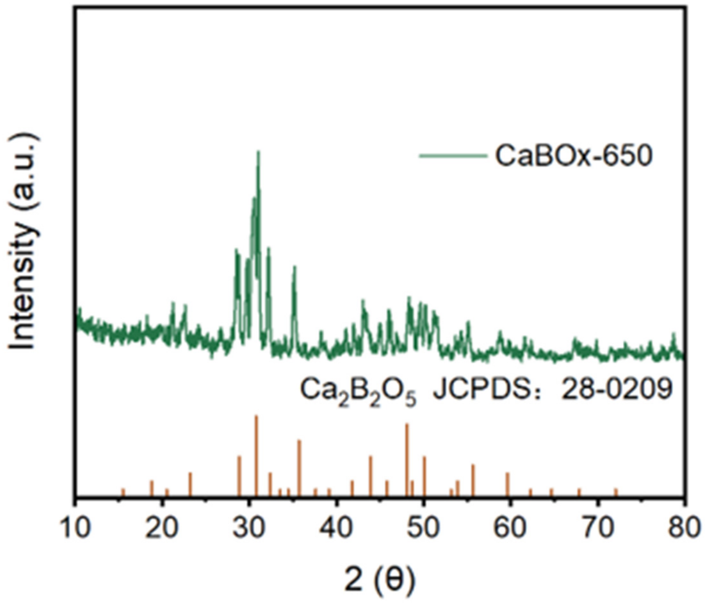

Figure S6. X-ray diffraction patterns of CaBOx-650.

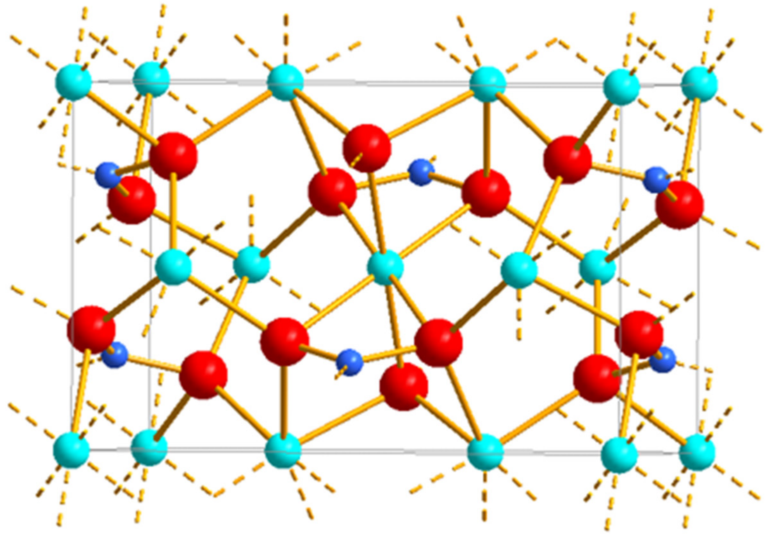

Figure S7. Structural diagrams of catalyst NiBOx-1000.

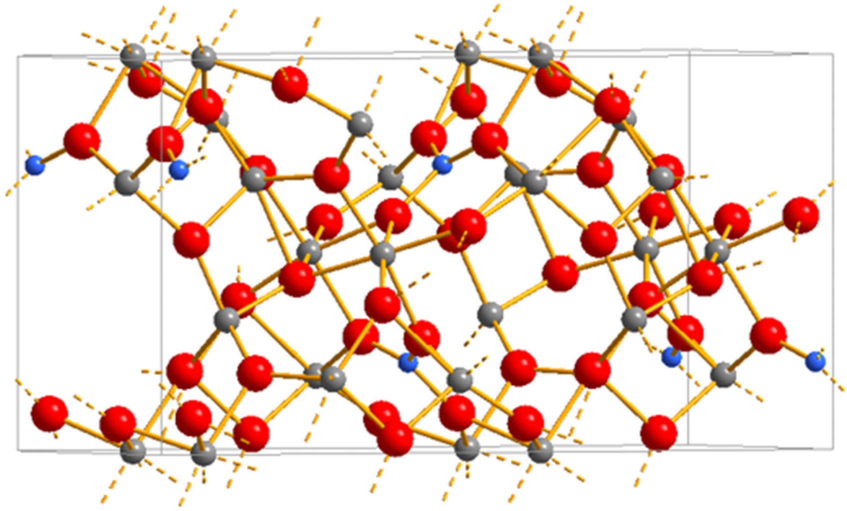

Figure S8. Structural diagrams of catalyst AlBOx-1000.

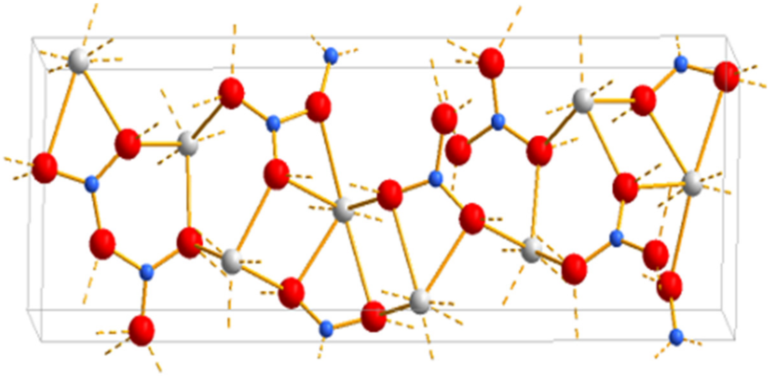

Figure S9. Structural diagrams of catalyst CaBOx-1000.

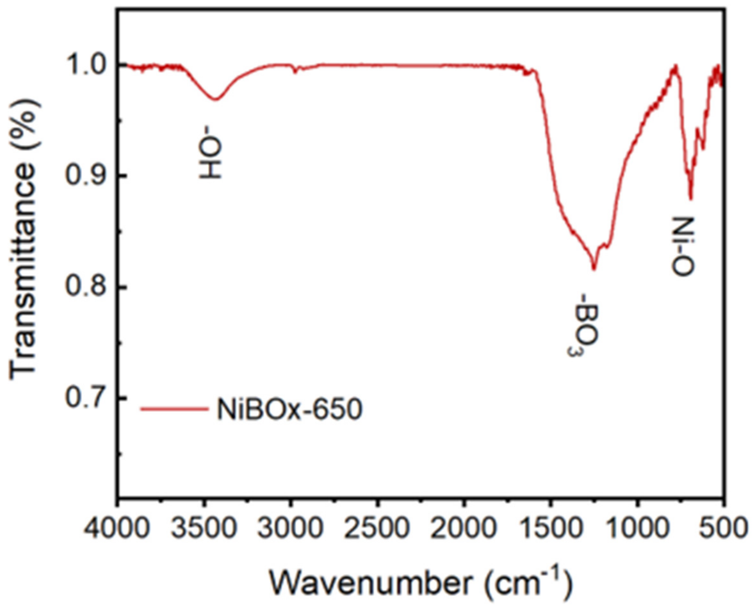

Figure S10. Fourier transform infrared (FTIR) spectroscopy of catalyst NiBOx-650.

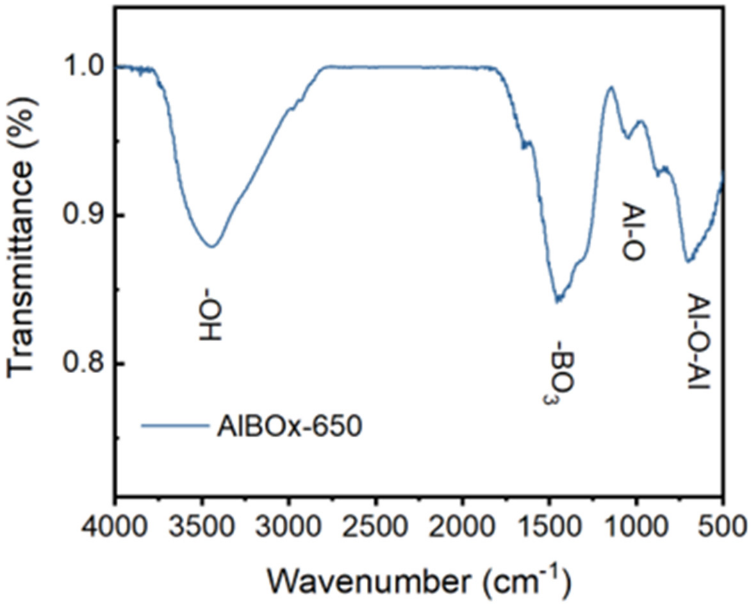

Figure S11. Fourier transform infrared (FTIR) spectroscopy of catalyst AlBOx-650.

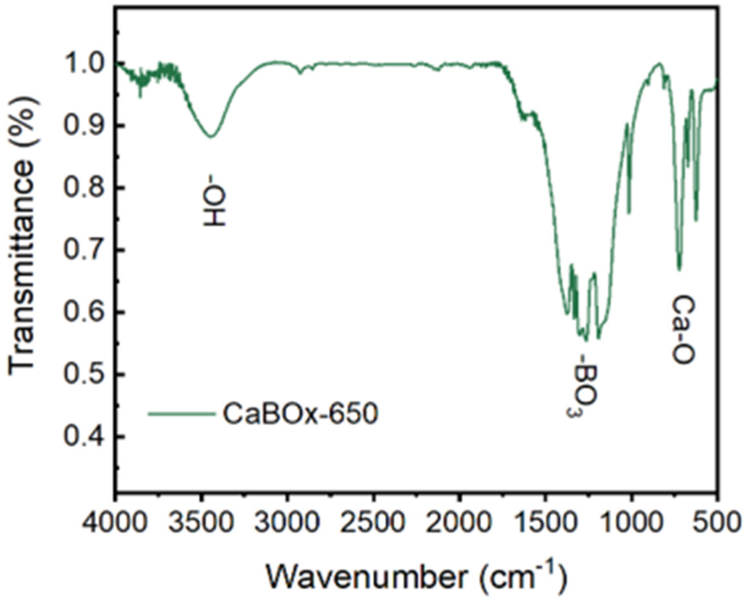

Figure S12. Fourier transform infrared (FTIR) spectroscopy of catalyst NiBOx-650.

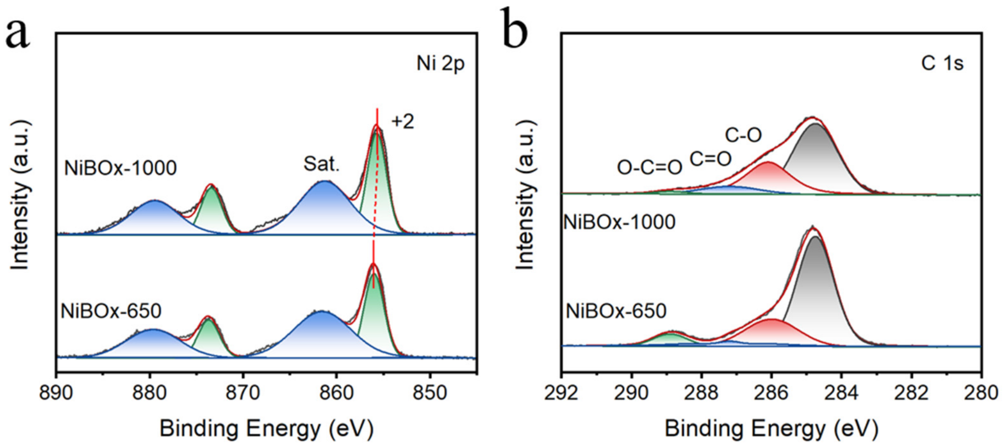

Figure S13. (a) Ni 2p and (b) C 1s X-ray photoelectron spectroscopy (XPS) of borate catalyst NiBOx-650 and NiBOx-1000.

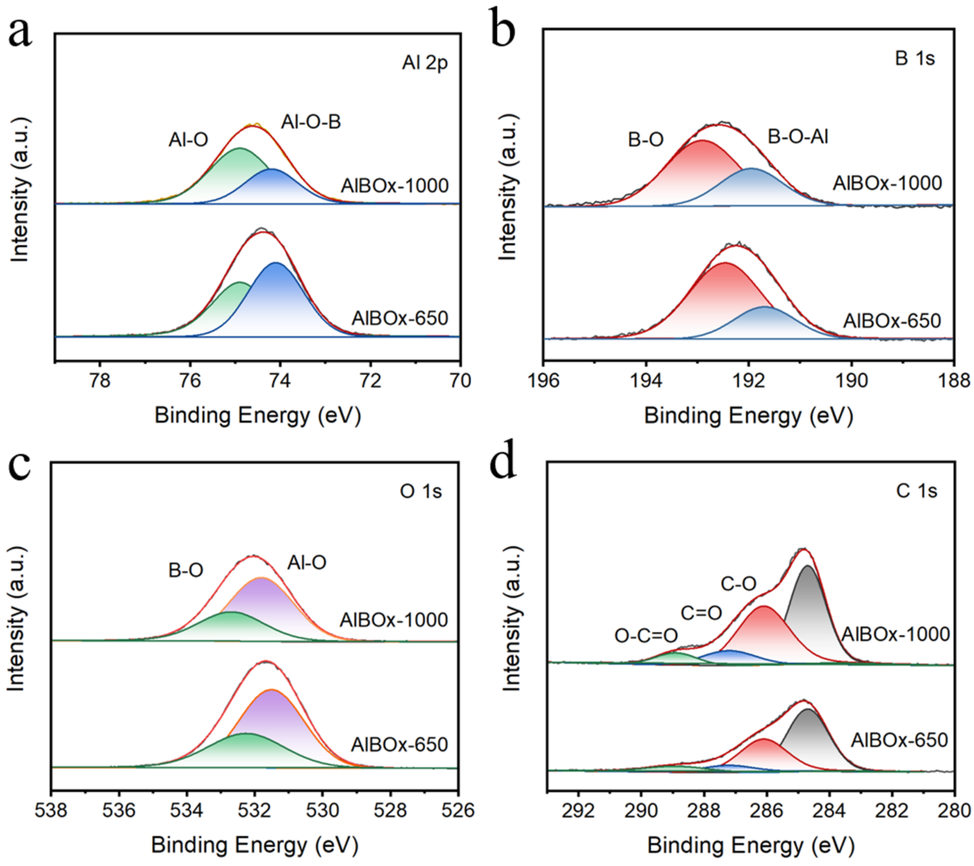

Figure S14. (a) Al 2p, (b) C 1s, (c) O 1s, and (d) C 1s X-ray photoelectron spectroscopy (XPS) of borate catalyst AlBOx-650 and AlBOx-1000.

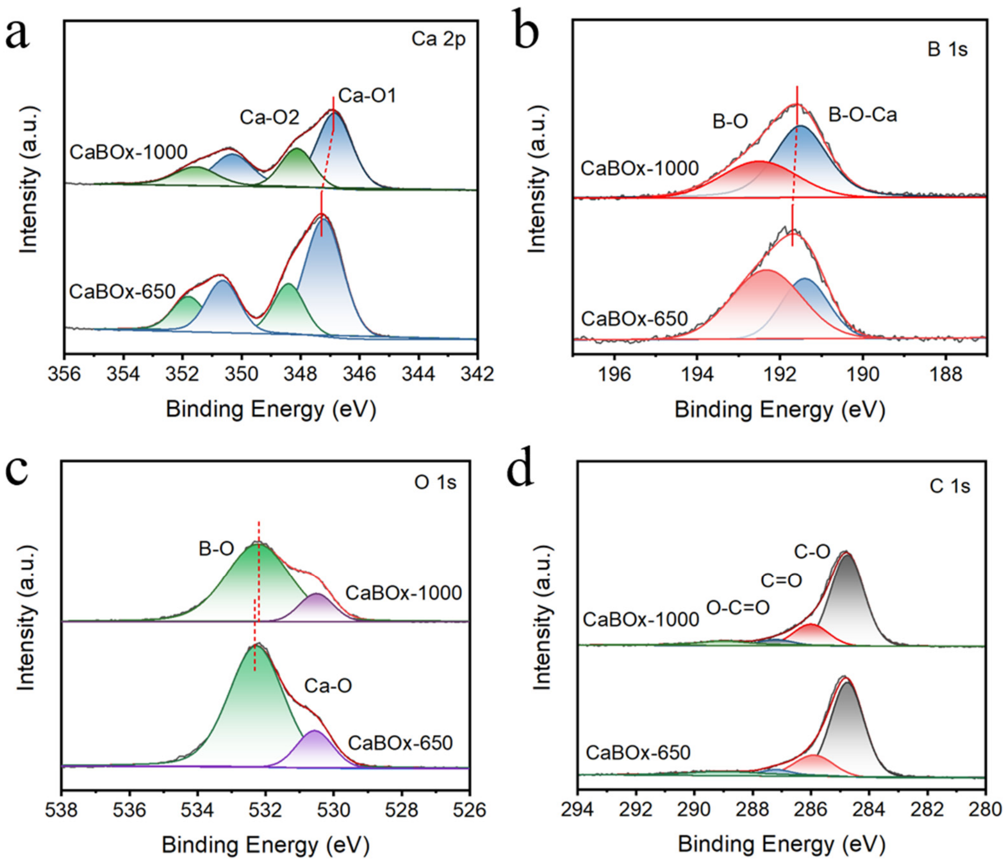

Figure S15. (a) Ca 2p, (b) C 1s, (c) O 1s, and (d) C 1s X-ray photoelectron spectroscopy (XPS) of borate catalyst CaBOx-650 and CaBOx-1000.

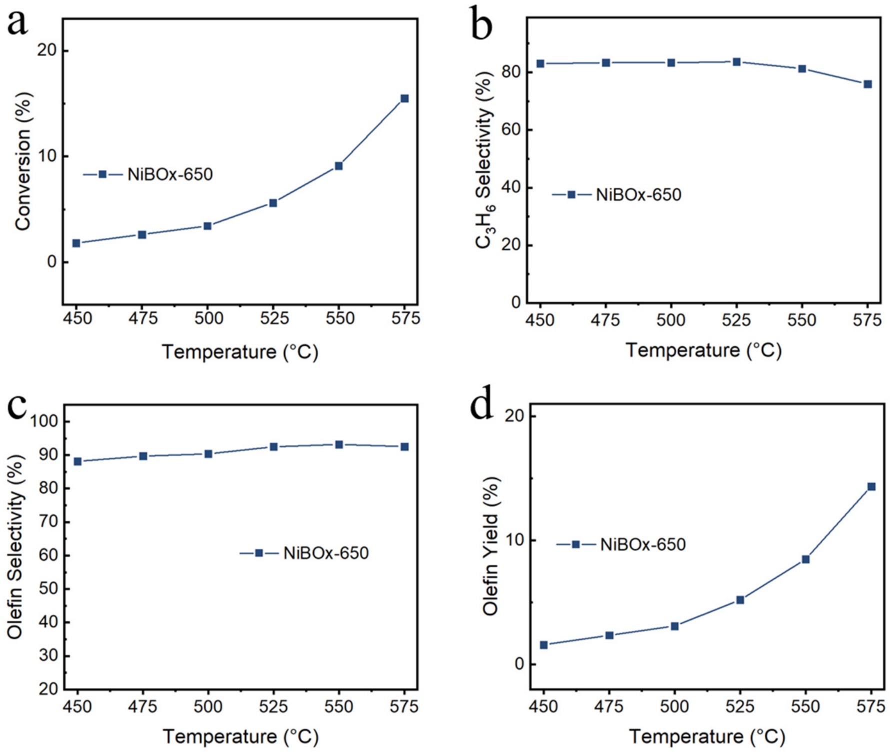

Figure S16. Propane oxidative dehydrogenation performance of borate catalysts NiBOx - 650, (a) Propane conversion, (b) C_3H_6 selectivity, (c) Olefin selectivity, (d) Olefin yield.

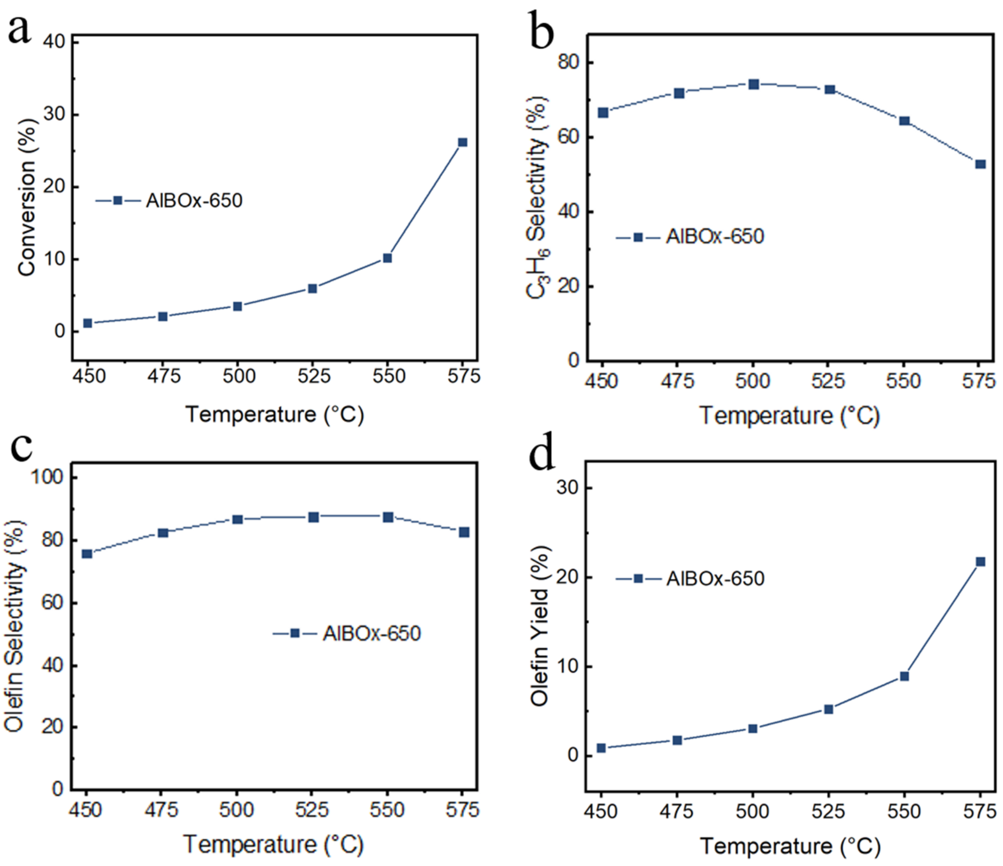

Figure S17. Propane oxidative dehydrogenation performance of borate catalysts AlBOx -650, (a) Propane conversion, (b) C_3H_6 selectivity, (c) Olefin selectivity, (d) Olefin yield.

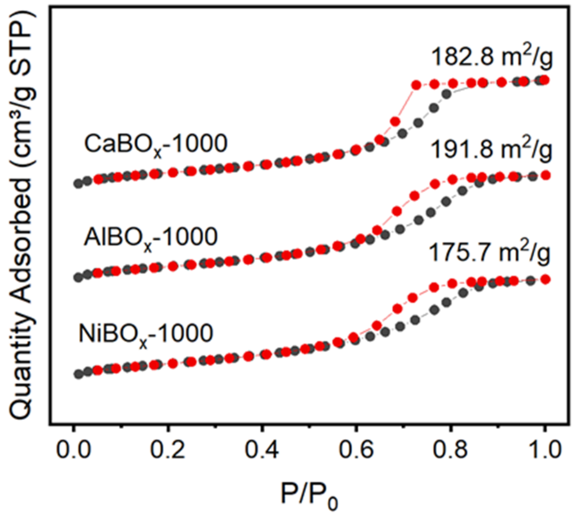

Figure S18. N_2 adsorption-desorption isotherms of the borate catalysts.

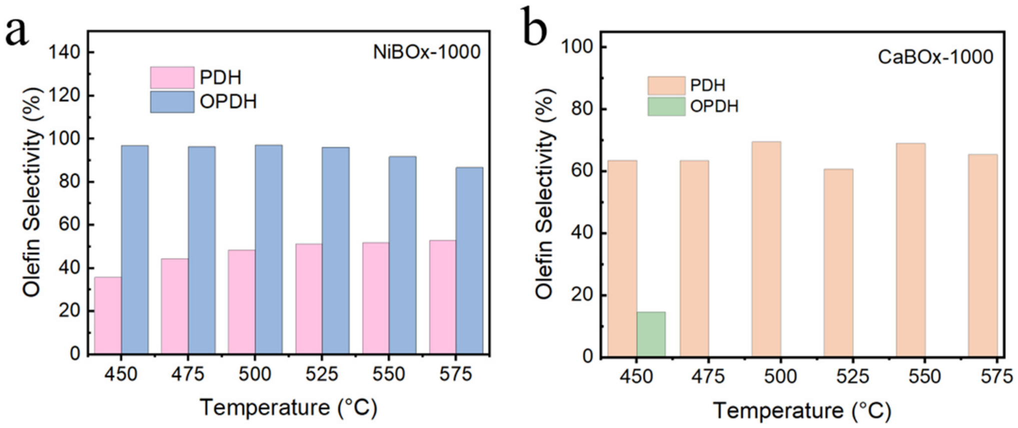

Figure S19. Catalytic performance comparison of propane oxidative dehydrogenation and direct dehydrogenation over the catalyst (a) NiBOx-1000 and (b) CaBOx-1000.

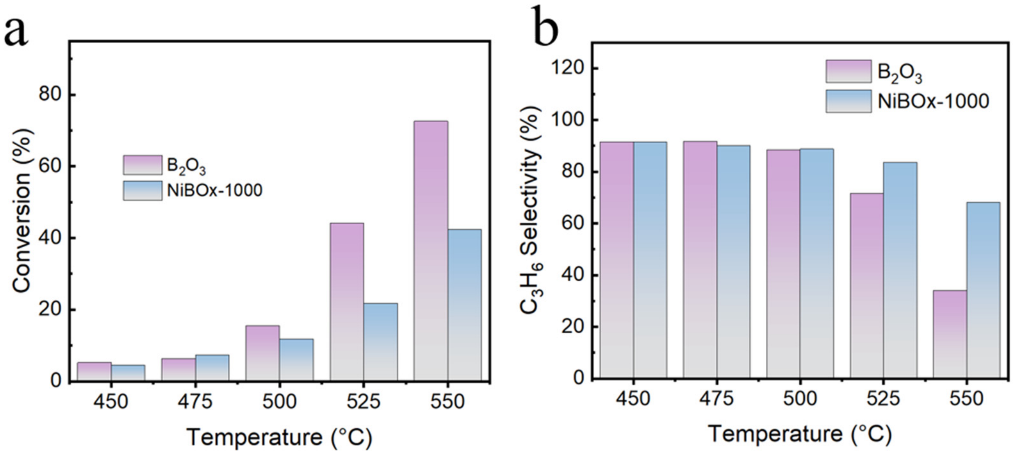

Figure S20. Propane oxidative dehydrogenation performance of catalyst NiBOx-1000 and B_2O_3: (a) Propane conversion, (b) Olefin selectivity.

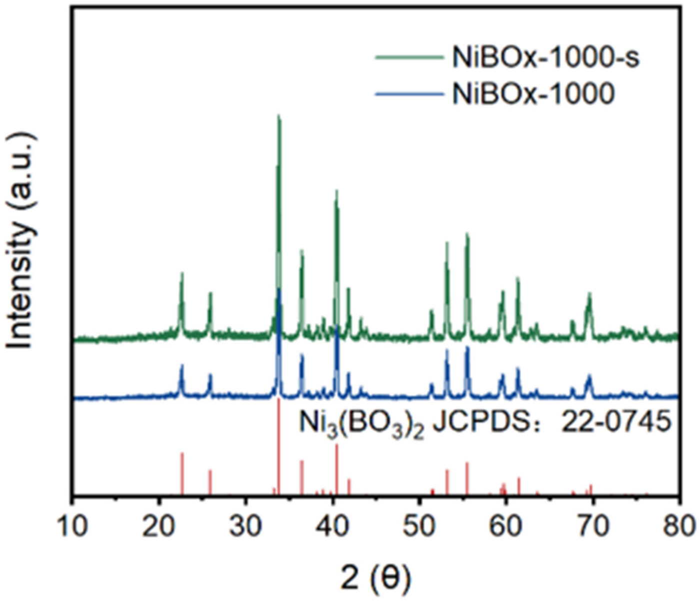

Figure S21. X-ray diffraction patterns of fresh and spent NiBOx-1000.

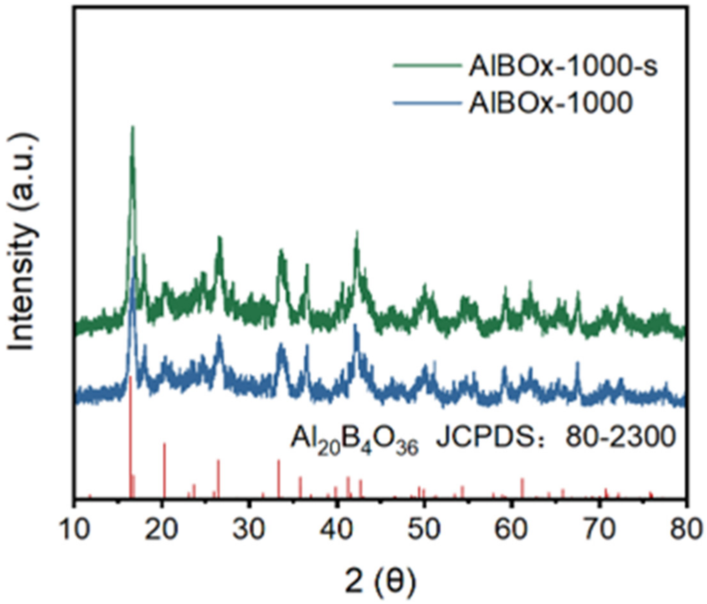

Figure S22. X-ray diffraction patterns of fresh and spent AlBOx-1000.

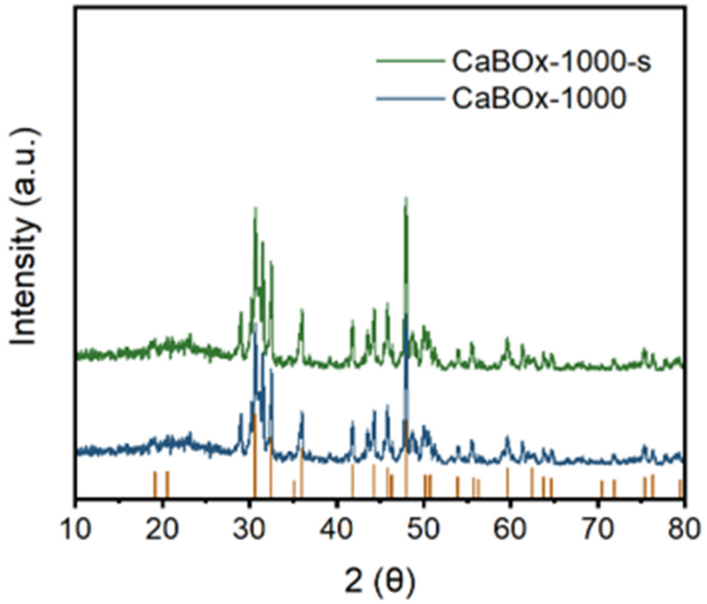

Figure S23. X-ray diffraction patterns of fresh and spent CaBOx-1000.
